# Key oncogenic signaling pathways affecting tumor-infiltrating lymphocytes infiltration in hepatocellular carcinoma: basic principles and recent advances

**DOI:** 10.3389/fimmu.2024.1354313

**Published:** 2024-02-15

**Authors:** Xiang Wang, Zijun Yuan, Zhengbo Li, Xinyu He, Yinping Zhang, Xingyue Wang, Jiahong Su, Xu Wu, Mingxing Li, Fukuan Du, Yu Chen, Shuai Deng, Yueshui Zhao, Jing Shen, Tao Yi, Zhangang Xiao

**Affiliations:** ^1^ Laboratory of Molecular Pharmacology, Department of Pharmacology, School of Pharmacy, Southwest Medical University, Luzhou, China; ^2^ Department of Laboratory Medicine, The Longmatan District People’s Hospital, Luzhou, China; ^3^ Cell Therapy and Cell Drugs of Luzhou Key Laboratory, Luzhou, Sichuan, China; ^4^ South Sichuan Institute of Translational Medicine, Luzhou, Sichuan, China; ^5^ School of Chinese Medicine, Hong Kong Baptist University, Hong Kong, Hong Kong SAR, China

**Keywords:** tumor-infiltrating lymphocytes (TILs), hepatocellular carcinoma (HCC), immunotherapy, signaling pathway, TILs preparation, targeting neoantigens

## Abstract

The incidence of hepatocellular carcinoma (HCC) ranks first among primary liver cancers, and its mortality rate exhibits a consistent annual increase. The treatment of HCC has witnessed a significant surge in recent years, with the emergence of targeted immune therapy as an adjunct to early surgical resection. Adoptive cell therapy (ACT) using tumor-infiltrating lymphocytes (TIL) has shown promising results in other types of solid tumors. This article aims to provide a comprehensive overview of the intricate interactions between different types of TILs and their impact on HCC, elucidate strategies for targeting neoantigens through TILs, and address the challenges encountered in TIL therapies along with potential solutions. Furthermore, this article specifically examines the impact of oncogenic signaling pathways activation within the HCC tumor microenvironment on the infiltration dynamics of TILs. Additionally, a concise overview is provided regarding TIL preparation techniques and an update on clinical trials investigating TIL-based immunotherapy in solid tumors.

## 1 Introduction

Liver cancer, the most common type of liver tumor, is one of the six most common cancers in the worldwide. It is ranked fourth in cancer deaths (1). Primary liver cancer encompasses hepatocellular carcinoma (HCC), intrahepatic cholangiocarcinoma, and other rare histological subtypes. The incidence rate of HCC is the highest among them, accounting for approximately 80% of all primary liver cancers. Furthermore, its incidence rate shows a progressive increase each year (1). The treatment modalities for HCC encompass surgical resection, liver transplantation, image-guided ablation, radiation therapy, transarterial chemoembolization (TACE), Chinese herbal medicine, and other interventions (2, 3). However, the recurrence rate of HCC after surgical resection is high, approximately 70% (4). Additionally, HCC is mostly found to be advanced or metastatic at the time of detection, with limited surgical resection pointers (5). The aforementioned statement suggests that the therapeutic options for HCC are limited, necessitating the exploration of alternative combination therapies. In recent years, tumor immunotherapy has emerged as a prominent field in scientific research. The primary mechanism of immunotherapy is to stimulate the immune system for targeted elimination of tumor cells, thereby achieving effective control over liver cancer. The primary drugs utilized for immunotherapy for HCC are immune checkpoint inhibitors (ICI), specifically anti-cytotoxic T-lymphocyte antigen 4 (CTLA-4) and anti-programmed cell death 1 (PD-1/PD-L1) monoclonal antibodies, which have been extensively employed in the treatment of HCC (6). Despite their efficacy, drug resistance to ICIs in treating HCC is prevalent, often due to various factors like deficient antitumor T cells and impaired memory T cells formation (7). The field of adoptive cell therapy (ACT) encompasses three primary modalities: tumor-infiltrating lymphocytes (TIL), genetically engineered T cell receptor (TCR), and chimeric antigen receptor (CAR) T cells (8, 9).In contrast to ICI, which inhibits T-cell suppressor receptors, ACT relies on *in vitro* culture and expansion of genetically modified or engineered T cells, thereby enhancing T-cell specificity (10).

TIL therapy involves obtaining tumor tissue from a patient, then isolating the infiltrating lymphocytes and selecting tumor-responsive cells from it. These cells are cultured and expanded *in vitro* and then returned to the patient. TIL therapies exhibit unique advantages in treating solid tumors due to their diverse TCR clonality, excellent tumor homing ability, and low off-target toxicity compared to other adoptive cell therapies (11). Rosenberg and colleagues conducted pioneering research in this field by demonstrating the *in vivo* patient-specific antitumor activity of cultivated TILs, starting from the 1980s (12). TILs therapy has shown to be beneficial in the treatment of solid tumors such as melanoma (13), breast cancer (14), and head and neck squamous carcinoma (15), etc. The therapy has also been successfully applied to liver cancer, and a clinical trial of TILs for primary HCC revealed higher patient survival rates and reduced side effects, thus demonstrating the reliability and safety of TIL therapy (16).

## 2 Tumor infiltrating lymphocytes

**Figure 1:**
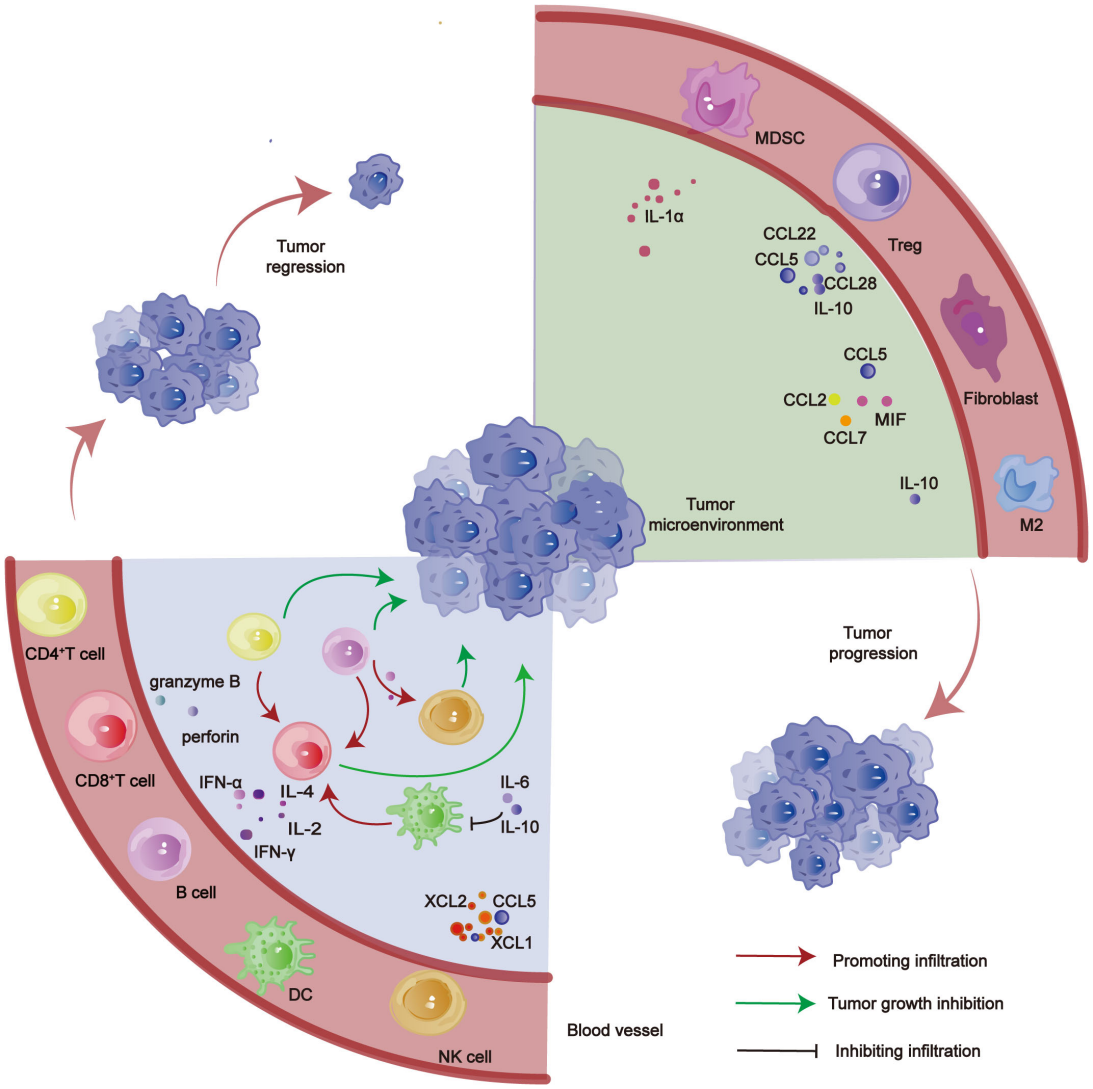
Role of various TILs in HCC.CD4^+^ T cell, CD8^+^ T cell, B cell, DC and NK cell inhibit tumor progression. MDSC, Tregs, Fibroblast, M2 and other cells promote tumor progression.

In HCC, TILs predominantly consist of various immune cells, including CD8^+^ T cells, CD4^+^ T cells, Tregs, TAMs, tumor-associated neutrophils, myeloid-derived suppressor cells (MDSCs), and NK cells (**Figure 1**). These immune cells play a pivotal role in the genesis and progression of HCC (17–20). The type and density of these infiltrating immune cells in tumors correlate with the prognosis of patients with HCC; a higher density of TILs infiltrating the tumor tissue is associated with longer survival periods (21, 22).

### 2.1 T lymphocytes

The tumor tissue in cellular carcinoma contains a specific number of TILs, and CD3^+^ T lymphocytes possess TCRs that recognize antigens presented by major histocompatibility complex (MHC) molecules on the surface of cancer cells. The presence of a high density of TILs in HCC has been implicated in the development of inflammation associated with HCC recurrence (23). However, in contrast to the findings of the previous complaint, another study with a limited sample size demonstrated a significantly reduced rate of tumor recurrence in patients diagnosed with HCC exhibiting high infiltration density of CD3^+^ and CD8^+^ cells in one or two samples (23). The findings of another meta-analysis, which included a cohort of over 1400 patients with HCC, demonstrated that a high infiltration of CD3^+^ T lymphocytes is associated with improved survival rates in HCC patients. Additionally, these TILs can serve as valuable prognostic indicators for evaluating patient outcomes (24).

The role of CD3^+^ T lymphocytes in HCC remains a subject of controversy and warrants comprehensive exploration and investigation. The involvement of CD3^+^ T lymphocytes in HCC is intricate and multifaceted, encompassing both patient recurrence and survival rates. A thorough comprehension and targeted examination of the interactions between tumor cells and CD3^+^ T lymphocytes are imperative for the advancement of efficacious immunotherapies for HCC.

#### 2.1.1 Infiltrating CD8^+^ T cells: density affects tumoricidal efficacy

CD8^+^ T cells, extensively studied star cells, play a crucial role in combating cancer by recognizing MHC-I antigen peptides on cancer cells through the TCR on their cell surface and releasing perforin and granzymes to eliminate tumor cells (**Figure 1**). To elucidate the disparities between immune cells derived from healthy liver tissues and those obtained from HCC patients, Nataliya Rohr-Udilova et al. conducted a comprehensive investigation encompassing immune cell populations sourced from over 40 individuals with normal liver function and more than 300 HCC patients (25). The analysis revealed that the predominant subset of infiltrating T lymphocytes in patients with HCC consisted primarily of CD8^+^ T lymphocytes, followed by CD4^+^ T lymphocytes (25). The ability to selectively isolate CD8^+^ T cells exhibiting medium to high infiltration within HCC tissue is believed to exert a favorable impact on patient prognosis (24). The surface marker CD103 is considered crucial for identifying tumor-infiltrating CD8^+^ T lymphocytes, and HCC patients with elevated levels of CD103-expressing CD8^+^ T cells exhibit improved survival rates compared to those with lower expression of this infiltrating cell population (26). These studies have demonstrated that CD8^+^CD103^+^ TILs possess the ability to secrete inflammatory cytokines, such as interferon gamma (IFN-γ) and tumor necrosis factor alpha (TNF-α), which play a crucial role in orchestrating the immune response of the body (27, 28). The tumor microenvironment facilitates the expression of E-calmodulin by epithelial cancer cells, which in turn interacts with CD103 on CD8^+^ T cells, thereby promoting the infiltration and persistence of tumor antigen-reactive CD8^+^ T cells within tumor tissues and sustaining their antitumor effects (29). The density of CD8^+^ T lymphocytes infiltrating tumor tissue may be influenced by the transcription factor (TF) Runx3, which is among the factors impacting their aggregation or depletion in HCC. In a mouse model of melanoma, it was observed that CD8^+^ TIL lacking Runx3 exhibited reduced aggregation within tumor tissue, resulting in accelerated tumor progression and unfavorable prognosis. Conversely, elevated expression of Runx3 led to increased density of TIL in tumor tissue and improved tumor prognosis (27). Depletion of tumor endothelial cells (TECs) through the expression of glycoprotein nonmetastatic melanoma protein B (GPNMB) induces infiltration of CD8^+^ T cells into HCC tissue (30). It is also noteworthy that the co-stimulation of 4-1BB and anti-4-1BB agonistic antibodies results in enhanced functionality of CD8^+^ TILs in patients with HCC (30). Moreover, CD8^+^ TILs exhibit a heightened activation status and increased tumor responsiveness, thereby rendering their impact on tumor control more enduring. This discovery opens up new avenues for the treatment of HCC (30). The current study revealed that only a minor subset of infiltrating lymphoid CD8^+^ T cells possess tumor recognition capabilities, while the majority remain non-functional. Therefore, it is crucial to identify and isolate this small fraction of highly cytotoxic cells (31). The identification of the tumor-specific marker CD39 in T cells enabled us to differentiate between CD8^+^ T cells specific to the tumor and those involved in paracrine signaling (32). The high-affinity neoantigen (HAN) disease-specific CD8^+^T cells were identified in the CD8^+^CD39^+^ TILs from HCC. Moreover, the anti-tumor activity exhibited by the high HAN group surpassed that of the low HAN group, which demonstrated a significant association with prognosis in HCC (33).

The balance between positive and negative effects of CD8^+^ T cells within the HCC microenvironment is complex and can be influenced by various factors. Understanding these interactions is crucial for developing effective immunotherapeutic strategies to target HCC and improve patient outcomes.

#### 2.1.2 Infiltrating CD4^+^ T cells: direct or auxiliary roles in immune cell-mediated tumoricidal actions

In 1986, Mosmann et al. published a groundbreaking article delineating the CD4^+^ Th cell population as a heterogeneous subpopulation (34). The investigations within this seminal study unveiled disparities in cytokine class production and functional attributes among CD4^+^ Th clones, thereby distinguishing distinct subpopulations of cellular antigens that elicit disparate immune responses, thus demarcating the Th1 and Th2 subsets (34). Subsequently, scientists have discovered immune-regulated factors and classified CD4^+^ T cells into distinct subpopulations such as Th3, Tregs, Tr1, Th17, Th9, and Th22 under varying immune regulatory effects. These subpopulations interact with each other to maintain immune homeostasis (35). The presence of infiltrating CD4^+^ T cells has been observed in various solid tumor types. Studies on head and neck squamous cell carcinoma and colorectal cancer tumor tissues have revealed that the co-expression of PD-1 and ICOS on solid tumors identifies CD4^+^ T cells reactive to the tumor (36). Additionally, these co-expressed CD4^+^ T cells recognize both tumor-associated antigens and tumor-specific neoantigens, thereby contributing to tumor immunity (36). The significance of CD4^+^ T lymphocytes in tumor regression is particularly noteworthy, and the dysregulation of lipid metabolism leading to the loss of CD4^+^ T cells in Non-alcoholic fatty liver disease (NAFLD) can expedite the progression of HCC (37). The absence of CD4^+^ T cells can impede the eradication of tumor cells, and research has demonstrated that inactivation of the oncogene *MYC* can recruit CD4^+^ T cells to the tumor site and induce sustained regression of tumors (38). The presence of infiltrated cytotoxic CD4^+^ T cells in tumor tissue decreases as HCC progresses, with peaks observed only during the early stages of the disease. Furthermore, a decrease in CD4^+^ cytotoxic T lymphocyte (CTL) levels is indicative of reduced survival time and increased mortality rates among patients with HCC (39).

The role of CD4^+^ T cells in promoting the generation of effector CD8^+^ T cells for tumor immunity has garnered significant attention, as evidenced by extensive research (40). However, recent research has revealed that CD4^+^ T cells infiltrating bladder cancer have the ability to express and destroy their own tumors in a manner consistent with MHC-II class presentation (41) (**Figure 1**). The cytotoxicity of CD4^+^ T cells enables them to directly target tumor cells independent of CD8^+^ T cell assistance (42). Additionally, transferred CD4^+^ T cells can effectively regulate tumor growth, either in isolation or through collaborative interactions with other immune cell populations (42). The CD4^+^ T lymphocytes have the ability to interact with TAMs within the tumor microenvironment, thereby influencing the polarization of TAMs and shifting their phenotype towards a more anti-tumor M1-like state (43). This ultimately facilitates an effective immune response against HCC.

In summary, the impact of tumor-infiltrating CD4^+^ T lymphocytes on HCC progression is intricate and contingent upon the context. The equilibrium among different subsets of CD4^+^ T cells, their cytokine production, and interactions with other immune cells in the tumor microenvironment collectively determine whether the immune response favors tumor growth or inhibits it. Exploiting the potential of CD4^+^ T lymphocytes and manipulating their functions could potentially serve as a therapeutic strategy to enhance the immune response against HCC.

#### 2.1.3 Infiltrating Tregs: modulating infiltrations in cytotoxic T cells and other immune cells

Tregs can be divided into three distinct sets, the first being tTregs produced in the thymus with high affinity for self-antigen (44), the second being iTregs generated by naive T cells stimulated by TGF-β and interleukin (IL)-2 (45, 46), and the third being pTregs secreted by peripheral mature CD4^+^ helper T cells upon antigen encounter (46). The Tregs possess the capacity to suppress autoantigenic responses, and the infiltration of Tregs into tumor tissue may impede the antitumor response of tumors (47, 48). A consequence of depleting Tregs in the immune response against tumors is observed. There is evidence suggesting that targeting CD25 leads to depletion of tumor-infiltrating Tregs, resulting in an anti-tumor effect (49). A study revealed a potential association between Tregs in blood, normal tissues, and tumor infiltration, suggesting a possible correlation between Tregs in normal tissues and those present in tumor-infiltrated tissues. Both types of cells exhibited heightened activation states, indicating that the tumor tissue infiltrating Tregs were likely recruited from peripheral tissues (50, 51). Therefore, targeting CD25 may have an effect on normal tissue Tregs while depleting infiltrating Tregs, thus possibly producing an anti-tumor response along with autoimmunity (52). The identification of specific targets for tumor-infiltrating Tregs holds paramount significance in the context of antitumor therapy.

**Figure 2:**
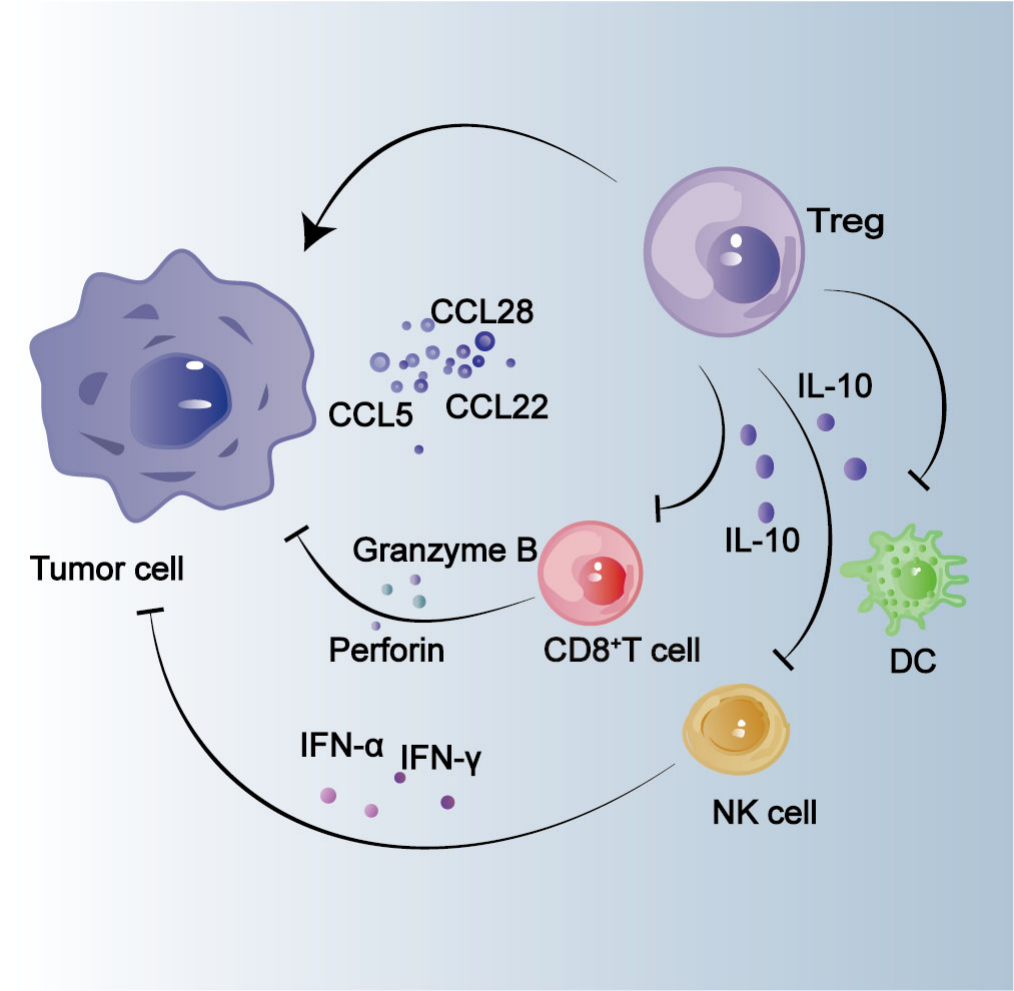
Effect of Tregs on tumor cells. Tumor cells secrete chemokines such as CCL22, CCL28 and CCL5 to recruit Tregs and promote tumor progression. IL-10 secreted by Tregs inhibits the infiltration of CD8^+^ T cells, DCs, and NK cells, indirectly promoting tumor progression.

The analysis of HCC tissues from 25 patients revealed a higher abundance of CD4^+^ CD25^+^ T cells in HCC compared to normal tissues (**Figure 1**) (53). Furthermore, the presence of this specific cell subpopulation may exert suppressive effects on the activity of CD8^+^ cytotoxic T cells, thereby contributing to the progression of HCC (53). As previously mentioned, Tregs, a subpopulation of CD4^+^ T lymphocytes, can be induced by HCC cells to secrete chemokines such as C–C motif chemokine ligand (CCL) 5,CCL22, CCL28, etc., which subsequently promote the accumulation of Tregs (**Figure 2**) (54–60). The findings of the study revealed that IL-8 secretion by HCC cells facilitates lactic acid accumulation, thereby augmenting the infiltration of Tregs (61). The complementary synergy arises from the accumulation of Tregs, which subsequently promotes the development of HCC (62–64). Tregs have the ability to secrete various suppressive cytokines, including IL-10 and TGF-β, which are known to inhibit the activation of CTL and Th1 CD4^+^ T cells. This ultimately diminishes the immune system’s killing effect and contributes to tumor development persistence (65, 66). The presence of other tumor-associated immune cells in the tumor microenvironment, along with the secretion of inducible factors by this population of immune cells, leads to the aggregation of CD4^+^ CD25^+^ Tregs, which play a crucial role in promoting tumor progression (67, 68). By analyzing the tumor tissues of 119 patients diagnosed with HCC, it was discovered that Tc17 cells, a subset of CD8^+^ T cells expressing IL-17, were present within the tumor tissues. Furthermore, IFNγ^−^Tc17 cells were identified as the predominant subgroup among the isolated Tc17 population (69). The presence of IFNγ^−^Tc17 was found to be associated with the progression of HCC, while CCL20 was shown to enhance the immunosuppressive capacity of IFNγ^−^Tc17 by promoting Tregs infiltration into tumor cells (69). The presence of tumor-infiltrating Tregs in HCC may impede the function of cytotoxic T cells and NK cells, thereby enabling immune evasion by the tumor and facilitating its growth (**Figure 2**) (45, 70).

It should be noted that the precise role of tumor-infiltrating Tregs in HCC progression can vary depending on the specific characteristics of the tumor, the patient’s immune profile, and other factors. Ongoing research is focused on unraveling the intricate interactions within the tumor microenvironment (TME) to develop targeted therapies that harness the immune system for combating HCC and other cancers.

### 2.2 Infiltrating B lymphocytes: auxiliary impacts on immune cell infiltration

B lymphocytes are also a constituent of the TILs. The interaction between T and B cells in the anti-tumor immune response has been well-established (71). The activation of cytotoxic and helper T cells (CTL and Th) is influenced by B cells, and depletion of B cells leads to reduced activation of CTL and Th (72). The value-added and survival of activated CTLs can be achieved through their binding to soluble CD19 secreted by CD27^+^ B cells, indicating a supportive role of B cells in the tumor immune response (73). B cells possess the capability to secrete cytokines IL-2 and IL-4, which facilitate the differentiation of helper T cells into Th1 and Th2 subsets, thereby actively participating in the immune response (**Figure 1**) (74, 75). B cells can also produce IFN-α and IFN-γ, thereby activating other immune cells such as NK cells and facilitating their involvement in tumor eradication (76). CD20^+^ B cells may serve as antigen-presenting cells (aAPCs), promoting T cell expansion and contributing to anti-tumor immunity (77). Inflammatory factors secreted by regulatory B cells (Bregs), including IL-10, IL-35, and TGF-β, can modulate T-cell activity (77). Moreover, Bregs possess the capability to recruit Tregs and promote tumor progression (78).

The impact of tumor-infiltrating B cells (TIBs) density on T lymphocyte infiltration in HCC has been demonstrated, while depletion of mature CD20^+^ B cells result in reduced local T cell activation and impaired tumor immunity (79). Subsequent investigations have revealed that the primary influence of B lymphocyte infiltration is on the expression of IFN-γ, GZB, and CD69 on CD4^+^ T lymphocytes, with a lesser effect observed on CD8^+^ T cells (79). Furthermore, Bregs directly interact with HCC cells to promote tumor growth and invasiveness through the CD40/CD154 signaling pathway (80).

In conclusion, TIBs play an important role in tumor immunity. In HCC for TIBs studies have also been progressing.

### 2.3 NK cell infiltration: direct tumoricidal actions and impacts on immune cell infiltration

NK cells are a critical subset of immune cells that primarily function through recognition and elimination of target cells, as well as secretion of cytokines such as IFN-γ and TNF-α to regulate the antiviral immune response (81). The presence of the inhibitory receptor NKG2A in tumors confers resistance to NK cell therapy (82). NKG2A/CD94 is an additional diverse receptor expressed on NK cells, facilitating the elimination of infected cells while preserving normal cellular function (83). Moreover, NK cells can recruit dendritic cells (DCs) to the tumor site by secreting chemokines such as XCL1, XCL2, and CCL5 (84, 85). These chemokines selectively bind to the XCR1 receptor expressed on conventional type 1 DCs (cDC1), playing a pivotal role in priming CD4^+^ and CD8^+^ T cell responses (84, 85).

Studies have demonstrated the presence of infiltrating NK cells in HCC tissues, wherein their expression is downregulated (86, 87). These NK cells can be recruited to the tumor microenvironment for direct elimination of tumor cells and promotion of dendritic cell recruitment and maturation. Moreover, the secreted cytokines also exert an influence on macrophage phagocytosis (**Figure 1**) (86, 87). The degree of infiltration by NK cells in HCC is positively associated with patient survival, while impaired NK cell function accelerates disease progression. Moreover, the presence of infiltrating NK cells enhances the effectiveness of sorafenib treatment for HCC (81, 88–90). Hepatocellular carcinoma-infiltrating NK cells exhibit distinct phenotypes that confer resistance to viral infection and promote antitumor activity. Additionally, these cells modulate the function of infiltrating T-cells to synergistically counteract HCC (91). The quantity of NK cells is positively correlated with the number of antigen-specific T cells, which are lymphocytes (92). NK cells can suppress antitumor responses by reducing the infiltration of T cells (92). Tumor-infiltrating NK cells are associated with Tregs, and an increase in CD4^+^ CD25^+^ Tregs may impair the ability of tumor-infiltrating NK cells to produce IFN-γ and eliminate K562 targets, thereby affecting their anti-tumor efficacy (**Figure 2**) (93). Due to their surveillance role and direct cytotoxicity against tumor cells, NK cells have potential as a target for immunotherapy in HCC treatment (91). Different subpopulations of infiltrating NK cells have been identified in HCC tissues, and the regulatory role of this specific subpopulation in HCC may be associated with the promotion of angiogenesis (94). GYT-303 has been designed based on the NK cell receptors NKp46 and Glypican-3 (GPC3), which have been experimentally demonstrated to mediate redirected killing of HCC cells by NK cells (95). The advantageous potential of NK cells in treating HCC was highlighted in a recent clinical study where hepatic artery infusion of NK cells resulted in a patient overall response rate (ORR) of 63.6% (96). Inhibition of NK cell activation is associated with IL-1α released from tumor tissues, which also attenuates the killing ability of CD8^+^ T cells; moreover, IL-1α released from tumors recruits MDSC in the tumor microenvironment and promotes HCC development (97).

Significant role in the context of cancer, including HCC progression. NK cells are a type of innate immune cell known for their ability to recognize and destroy abnormal cells, including cancer cells, without prior sensitization. Efforts to harness the potential of NK cells for cancer therapy, including HCC, are ongoing. Strategies include using NK cell-based therapies, combination therapies targeting immune checkpoints, and modifying the tumor microenvironment to promote NK cell activity. As research advances, a deeper understanding of these interactions could lead to more effective immunotherapeutic approaches for treating HCC.

### 2.4 Other immune cells

#### 2.4.1 DC: assisting T cell differentiation

The DCs are a type of antigen-presenting cell (APC) responsible for capturing, processing, and presenting antigens to T cells, thereby initiating an immune response(**Figure 1**) (98). DCs capture tumor-derived antigens in the liver and present them to T cells in lymphoid organs like lymph nodes. This presentation is crucial for activating tumor-specific T cells that can effectively target and eliminate cancer cells (98, 99). pDCs are a subset of DCs associated with autoimmunity and capable of producing IFN-I. The secretion of IFN-I and IL-12 by pDCs facilitates the polarization of CD4^+^ T cells into Th1 helper T cells, which in turn promotes the accumulation of CD8^+^ T cells involved in the subsequent anti-tumor response (100). The presence of infiltrating pDCs has been observed in HCC tissues, and a negative correlation has been found between the degree of pDC infiltration and survival in HCC (**Figure 2**) (100). Infiltrating pDCs may exert an influence on the function of infiltrating Tregs in HCC, ultimately impacting the prognosis of this disease (101). However, the function of DCs is modulated by the tumor microenvironment (102). Additionally, α-fetoprotein (AFP) secreted by hepatocytes influences the function of DCs and impacts HCC progression in various ways (103). Factors associated with the tumor microenvironment (IL-10, IL-6) hinder the differentiation of DCs, decrease their maturation, and suppress T cell activation (104).

#### 2.4.2 M2 Macrophage infiltration: facilitating cancer progression

Macrophages differentiate from monocytes and exhibit dynamic phenotypic changes in response to inflammation, tumor progression, and fibrosis. They can be categorized into two types: M1 and M2 macrophages (105). M1 macrophages possess both pro-inflammatory and anti-tumor properties, while M2 macrophages display an anti-inflammatory phenotype (106). In HCC, the predominant type of macrophage is the M2-like subtype, which has been implicated in promoting tumor growth (107). Macrophages may promote depletion of infiltrating T cells in the early stages of the tumor, leading to HCC progression(**Figure 1**) (108). The types of macrophages influence the trajectory of tumor progression. M1 macrophages can recruit T cells and NK cells by secreting IL-12 and (C-X-C motif) ligand (CXCL)10, while M2 macrophages and Tregs can secrete IL-10 to suppress the activation of T cells, NK cells, and DCs (109).

Macrophages exhibit a high degree of infiltration in HCC, where M2 macrophages and fibroblasts may activate TGF-β, accelerating the depletion of surrounding CD8^+^ T cells. This process diminishes the functionality of CD8^+^ T cells, thereby reducing their tumoricidal capacity (104, 110). Consequently, immune depletion is observed in HCC (104, 110). The transcription factor FOXO1 specifically targets TAMs, leading to a reduction in IL-6 secretion by these macrophages. This inhibition of IL-6/STAT3 signaling pathway effectively hampers HCC progression (111). Not only does TAM impact other TILs, but it also influences the expression of Tregs in tumors through two pathways: 1) Recruitment of Tregs via secretion of CCL22; and 2) Induction of naïve T-cells to aggregate and differentiate into Tregs (CCL18 and IL-10) (112).

## 3 TILs preparation

Rosenberg’s team conducted pioneering research in the field by demonstrating the antitumor activity of TILs in patients in 1980 (12). The investigation revealed that only a minority of lymphocytes infiltrating the tumor tissue are reactive to the tumor (tumor-reactive TILs), while other TILs may recognize antigens unrelated to the tumor (non-tumor-specific antigens) (31). ACT with TILs, a highly specific treatment tailored for each patient, hinges on the isolation and expansion of tumor-reactive TILs (113).This therapy involves extracting tumor tissues from patients, isolating the infiltrating lymphocytes, selecting the tumor-reactive cells, and then cultivating and expanding these cells *in vitro* before reinfusing them into the patient.

### 3.1 Separation of TIL

**Figure 3:**
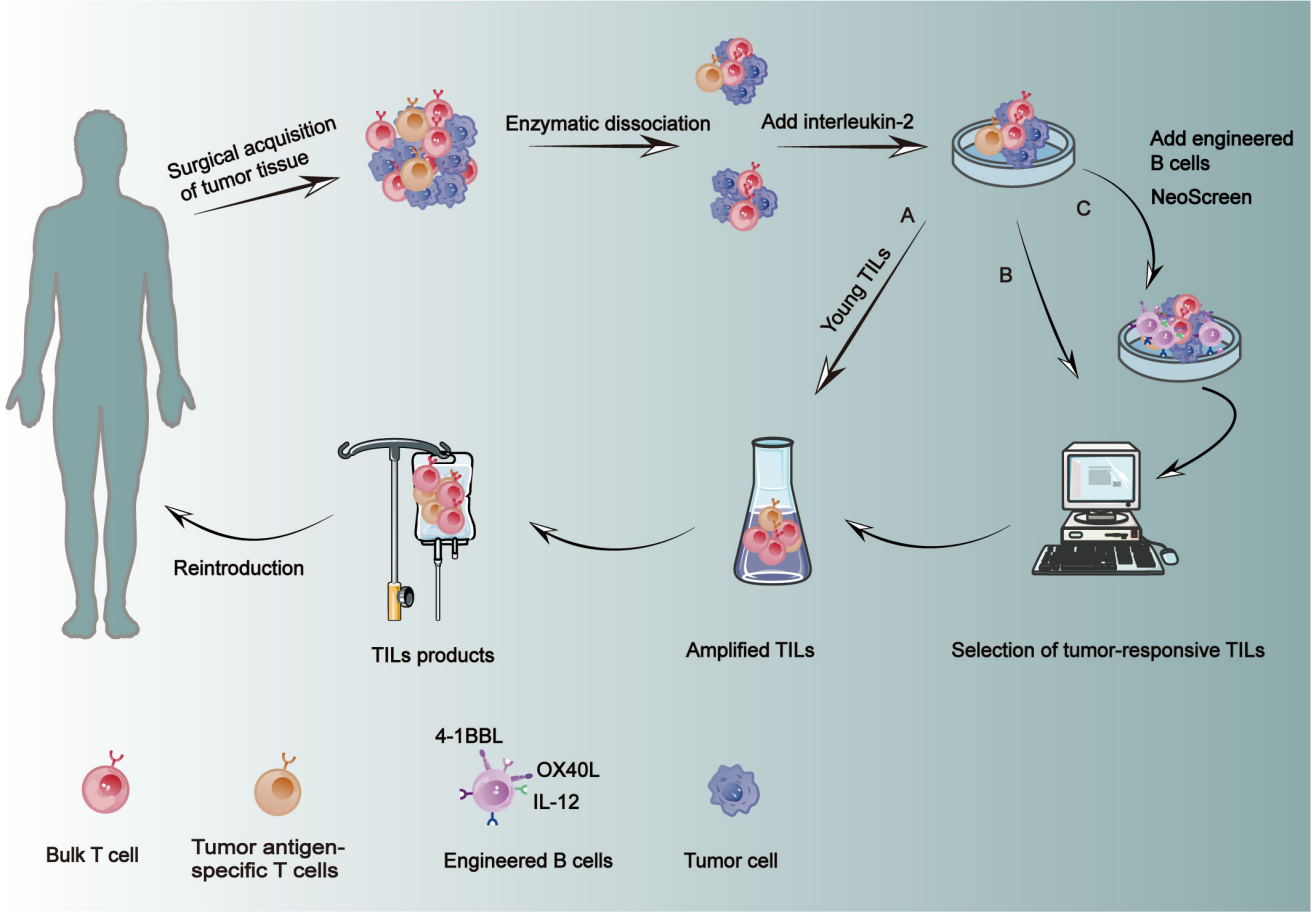
Preparation of TILs. Fresh tumor tissue was obtained from the patient and subjected to fragmentation enzymatic hydrolysis, adding IL-2 for standard amplification. **(A)** Young TILs: Secondary amplification is performed directly after standard amplification to shorten preparation time, and then transfused back into the patient. **(B)** Tumor-responsive TILs were selected for amplification and then transfused back into the patient. **(C)** NeoScreen: During amplification, the engineered B cells of CD40 were used as antigen presenting cells, and then the antigen specific T cells were amplified and transfused back to the patient. This method can significantly increase the frequency of specific TILs.

Obtaining the tumor tissue, typically through surgery, must be processed promptly to avoid cell death, with sources ranging from the tumor itself to metastatic lymph nodes or even peritoneal or pleural effusions (114–116). Tumor tissue is excised from the necrotic regions, while normal tissue is precisely sheared to the appropriate dimensions. Subsequently, an enzymatic digestion of a mixture containing collagenase, hyaluronidase, *etc.*, is performed overnight to generate a homogeneous single-cell suspension (**Figure 3B**) (117). The enzymatically digested single-cell suspensions should undergo filtration to eliminate undigested tumor tissue, followed by Ficoll density gradient centrifugation for the purpose of purification (117). The cell concentration should be quantified in order to determine the appropriate culture density (118).

### 3.2 Culture of TIL

In the pre-amplification phase, enzymatically isolated TIL or tumor tissue blocks of appropriate size are cultured in a specialized medium supplemented with a high concentration of IL-2 (119, 120). Primary cultures are typically established in 24-well plates, and the culture medium is regularly refreshed throughout the process, which usually spans a duration of 2-4 weeks (119, 120). The expanded TILs can be phenotyped using flow cytometry, while the Elispot assay enables quantification of effector T cells capable of releasing IFN-γ prior to proceeding with subsequent steps (**Figure 3B**) (121, 122). At this stage, a limited number of lymphocytes can be obtained, but the quantity is insufficient for retransfusion. Consequently, a high-dose expansion of lymphocytes is opted for. During the rapid expansion phase, TILs are transferred to culture flasks or permeable bags for cultivation in order to achieve a substantial cell yield. Subsequently, following the completion of the culture period, cells undergo concentration, washing, and suspension procedures to meet production specifications prior to their reinfusion (118). Conventional TIL culture methods necessitate the utilization of multiple containers and a substantial quantity of culture media and reagents, rendering them susceptible to microbial contamination and reliant on high staff expertise. To optimize the culture methodology, Jianjian Jin et al. employed permeable G-Rex bottles for TIL culture and expansion, resulting in reduced requirements for medium, reagents, and containers while maintaining comparable phenotype and cytokine production levels to those achieved with traditional methods (118).

Innovative TIL cultivation methods have emerged to precisely and rapidly obtain tumor-reactive TILs in the laboratory. In the pre-Rapid Expansion Protocol (pre-REP) phase, block-processed tumors are co-cultured with IL-2, CD3 antibodies, and 4-1BB antibodies to activate T cells, enhancing the success rate of TIL cultivation (121, 123, 124). This approach shortens the cultivation time and increases the number of expanded CD8^+^ TILs (123). Generally, TILs producing IFN-γ in co-culture with autologous tumor cells are considered tumor-reactive and are then expanded further, although this requires a longer cultivation time (125, 126). Rosenberg’s team introduced the “young” TIL method, eliminating the need for *in vitro* testing and tumor recognition of tumor fragments or digests (**Figure 3A**). The method rapidly expands the selected TILs, which may be antigen-reactive, thus reducing preparation time (125, 127). Another study showed that adding TGF-β during the REP phase could increase the percentage of CD8^+^ T cells in the TIL product without inhibiting overall T cell expansion (128). The key to enhancing the efficacy of TIL therapy lies in accurately selecting tumor-reactive T cells. The NeoScreen technology, which stimulates the expansion of TILs by loading tumor-associated antigens or neoantigens onto autologous aAPCs, offers new hope in this regard (**Figure 3C**). Unlike traditional TIL preparation methods, NeoScreen uses engineered B cells expressing CD40 (modified with OX40 ligand, IL-12 RNA, and 4-1BB ligand) as aAPCs in early culture stages to significantly increase the frequency of antigen-specific TILs, thereby expanding antigen-specific T cells (129, 130).

### 3.3 The return of TIL

The monitoring of residual tumor cells after TIL expansion can be achieved through the utilization of flow cytometry (FCM), immunohistochemistry (IHC), and fluorescence *in situ* hybridization (FISH) (131). Therapeutic products based on TILs are subject to stringent regulatory requirements ensuring their safety, purity, and potency. Prior to TIL administration, patients typically undergo lymphatic clearance as a means to enhance the therapeutic efficacy of this treatment approach.

Several immune cell populations, including MDSCs, Tregs, and TAMs, potentially influence the therapeutic efficacy of TIL by modulating T cell function (132). The nonmyeloablative clearance of lymphatics enhances the survival, persistence, and antitumor activity of TILs by eliminating Tregs, increasing intracellular homeostatic maintenance factors such as IL-7 and IL-15, and eliminating NK cells and myeloid cells that compete for these trophic factors (133). In addition, lymphatic clearance promotes CD8^+^ T cell proliferation and increased loss of tolerance to self-antigens, thereby enhancing the antitumor activity of T cells. At the same time, APCs have an increased cellular capacity, and lymphatic clearance leads to apoptosis that stimulates DC activation and promotes their migration to the lymph nodes, enhancing antigen-specific T cell responses (134, 135). The preferred mode of input is typically intravenous administration, although alternative modes such as intraperitoneal or intraperitoneal infusion may be utilized in select cases (136).

## 4 Targeting tumor specific neoantigens

In TIL therapy, precise targeting of neoantigens and *in vitro* expansion of T cells expressing specific TCRs, along with the maintenance of clonality in amplified T cells *in vivo*, is crucial for ensuring the specificity of TIL therapy. Dr. Rosenberg’s team has gained prominence by employing genetic engineering techniques or targeting surface markers.

### 4.1 Genetic engineering technology targets neoantigens

Previous studies have demonstrated that functional recognition of tumor-specific antigens by surface TCRs can be assessed *in vitro* cultured TILs. However, the non-specific expression on paracrine TILs may impact the accuracy of antitumor TIL recognition (137). The utilization of innovative peptides loaded with human leukocyte antigen (HLA) protein multimers to identify neoantigen-reactive T cells necessitates patient-specific investigations and is subject to certain limitations (137). In a recent study, researchers employed single-cell transcriptomics to analyze CD8^+^ T cells labeled with circulating neoantigens in peripheral blood. The utilization of the peripheral blood circulating neoantigen research approach offers enhanced convenience and reduced time consumption for investigating antitumor T cells and their TCR clonal phenotypes compared to invasive surgical methods required for obtaining TILs (137). However, given the existing technical and resource limitations, this study is still in its preliminary stages, necessitating further investigation to exclude the presence of non-antitumor-reactive T cells. The *TP53* gene is frequently mutated across various cancer types, and its encoded protein, p53, serves as a pivotal tumor suppressor gene that holds great potential for targeted immunotherapy against cancer (138). Studies investigating TIL and peripheral blood lymphocytes (PBL) have demonstrated the potential of T-cells to recognize *TP53*-mutated tumors, thereby presenting an opportunity for utilizing peripheral blood as a non-invasive therapeutic modality (139). In the context of this study, TIL with mutated p53 were cultured and subjected to *in vitro* screening. Subsequently, these modified TIL were reinfused into patients, resulting in the generation of neoantigen-responsive TIL through co-culturing with autologous APCs (140). Encouragingly, remission was observed in 2 out of 12 treated patients (140). However, TILs targeting neoantigens exhibit a limited presence of mutant p53-responsive T cells as well as diminished persistence (140). Researchers have also endeavored to clone TCRs responsive to p53 neoantigens into retroviral vectors for transduction into PBL derived from healthy donors, which has demonstrated superior therapeutic efficacy compared to TIL and is anticipated to streamline the treatment cycle by reducing the time required for neoantigen screening and T cell culture (140).

*RAS* oncogene mutations are observed in approximately 30% of cancer patients, and the successful targeting of TILs in *KRAS*-mutated colorectal cancer patients has been demonstrated in previous studies (141, 142). In recent studies, the feasibility of screening and stimulating TIL amplification using specialized *RAS* hotspot mutation reagents, or identifying and isolating other low-frequency *RAS*-responsive T cells in metastatic cancer patients’ PBL has been demonstrated (142). Previously, HLA-C*08:02-restricted TCRs targeting KRAS G12D were identified from TILs of patients with metastatic pancreatic cancer (143). These TCRs were engineered into autologous T cells and administered to a patient with metastatic pancreatic cancer, resulting in objective regression of the disease through this TCR gene therapy (143). This approach may offer new avenues for treating pancreatic cancer. By utilizing CRISPR, scientists were able to eliminate Cytokine-induced SH2 protein (CISH) from primary human TIL and observed that the resulting *CISH* deficiency led to an enhancement in TIL function as well as recognition of neoantigens (144).

In a recent clinical study, Rosenberg and his team administered mutation-reactive autologous lymphocytes to treat breast cancer, resulting in objective tumor regression observed in three out of six subjects (145). The method replaces tumor cells with tandem minigenes (TMGs) or peptides representing cancer mutations, eliminating the need to culture tumor cell lines and making it more convenient and faster (146). The specific steps involve identifying somatic mutations, constructing tandem minigenes and transfecting autologous APCs, co-cultivating tandem minigenes and peptide pools with autologous antigen-presenting DCs, and inducing T-cell competence for TILs capable of recognizing autologous mutations (146). The limited number of mutations in breast cancer and the small sample size prevent the assessment of both the abundance and number of neoantigenic responses, necessitating further studies (145).

### 4.2 Target neoantigens by surface markers

The enrichment of tumor-reactive T cells is achieved through the screening of T cells that co-express two or more dysfunctional markers and specific gene signatures. Surface marker-based sorting methods have the potential to enhance the effectiveness and efficiency of screening neoantigen-reactive TCRs from human TILs. Expression of the CD39CD69 marker correlated with T cell function, while stem-like CD8^+^ T cells were described as CD39^-^CD69^-^ T cells (147). The phenotype of anti-tumor TILs is characterized by the presence of CD39CD69; however, it should be noted that TILs currently exist in a terminally differentiated state and exhibit limited persistence in patients (147).

To expedite and enhance the screening process for neoantigen-reactive TILs, scientists isolated CD8^+^ T cell receptors linked to neoantigen responses from metastatic tumor digests by scrutinizing the single-cell transcriptomic profile of neoantigen-specific T-cell clonal phenotypes and identifying T-cells that co-express PD-1, CD39, and TIGIT markers (148). The proliferation and survival capacity of this method for screening new antigen-specific T cells *in vitro* is limited, thereby restricting its potential as a viable cell therapy product (148). Ken-ichi Hanada et al. employed CITE-seq (Cellular Indexing of Transcriptomes and Epitopes by Sequencing) and TCR-seq (TCR sequencing) technologies to examine fresh TILs in non-small cell lung cancer (149). They developed neoantigen-reactive T cell markers based on clonotype frequency, CD39 protein expression, and CXCL13 mRNA levels, and screened for neoantigen-reactive TCRs in both CD8^+^ and CD4^+^ T cells (149). The markers CD39 and CXCL13 have been identified as indicators of neoantigen-responsive T cells, facilitating the rapid identification of neoantigen-responsive TCRs and expediting the development of personalized therapies targeting neoantigens (149).

### 4.3 Machine learning models predict neoantigens

The prediction of neoantigen immunogenicity encounters the following challenges (150): a) Data limitation: The available neoantigen datasets are limited in size and exhibit bias, thereby failing to fully capture the diversity and degree of variation among neoantigens. b) Data quality: Existing datasets suffer from survival bias, sample source bias, and tumor type bias, which can potentially impact the training and predictive outcomes of models. c) Negative sample data: Supervised learning for neoantigen recognition necessitates negative sample data; however, technical and biological factors may introduce “false negatives,” wherein certain circumstances demonstrate immunogenicity for some neoantigens while others do not. d) Limited prediction scope: Current models predominantly focus on predicting CD8^+^ T cell responses, with less emphasis on predicting CD4^+^ T cell responses that pose greater challenges. To address these limitations, the researchers devised a novel approach integrating machine learning and bioinformatics techniques to enhance the predictive accuracy of neoantigen immunogenicity (150). This method was trained on a comprehensive dataset and its performance was validated through comparison with experimental data. The findings demonstrate a significant breakthrough in neoantigen immunogenicity prediction, offering crucial support for advancements in cancer immunotherapy (150). By employing machine learning models to analyze extensive sets of validated tumor neoantigen data, it becomes feasible to ascertain the specific mutant neoantigens that can be effectively recognized by T cells, thereby facilitating the targeted recognition and activation of TILs towards these neoantigens (151).

### 4.4 TIL therapy was predicted by combining immune features of HCC

The analysis of HCC revealed the presence of ten frequently mutated genes, namely *TP53*, *TTN*, *CTNNB1*, *MUC16*, *ALB*, *PCLO*, *MUC*, *APOB*, *RYR2* and *ABCA* (152). Notably, a high tumor mutational burden (TMB) was observed indicating substantial genomic heterogeneity in the tumor tissue (152). This finding may have implications for both patient survival and the extent of immune infiltration. PD-1 and TIGIT markers are expressed by depleted CD8^+^ T cells, which constitute a substantial proportion of HCC (153). They utilized whole exome sequencing (WES), RNA sequencing (RNA-seq), computational bioinformatics, and IHC to predict neoantigens in 22 patients diagnosed with HCC (154). Their findings revealed that patients harboring *TP53* neoantigens exhibited a significantly prolonged overall survival rate (154). Furthermore, they observed enhanced infiltration of cytotoxic lymphocytes and heightened immunocytolytic activity within the *TP53* neoantigen group (154).

We summarized the clinical trials of TIL therapy applied to solid tumors in the last 10 years (**Table 1**). The utilization of TILs is an emerging field in immunotherapy, offering a glimmer of hope for patients with solid tumors that have shown resistance to conventional immunotherapies. The application of TILs in HCC is currently being investigated through clinical trials. A total of 15 patients with primary HCC received TIL infusion following hepatectomy, resulting in disease-free status for 12 patients and tumor recurrence for the remaining three (16). All experiments have demonstrated the safety and efficacy of autologous TIL cell reinfusion. Another clinical trial on TILs in HCC (NCT04538313) is currently underway, focusing on the safety and tolerability of autologous TILs for treating high-risk recurrent primary HCC; however, the results of this trial have not yet been published. The field of TILs therapy for the treatment of HCC is continuously evolving, and efforts are underway to optimize treatment regimens, refine patient selection criteria, and explore combinations of therapies.

**Table 1:** Important clinical trial of TIL therapy for solid tumors.

Start Time	Disease	Clinical Trial	Method	Outcome	NCT
2010	Metastatic breast cancer	Phase II	Cyclophosphamide and fludarabine + young unselected tumor-infiltrating lymphocytes (TIL) + high-dose aldesleukin + pembrolizumab prior to cell administration	ORR	NCT01174121
2012	HPV-associated tumors	Phase II	Infusion of young TILs and injection of interleukins after a protocol for non-clear myeloid lymphoid failure	ORR (24%)	NCT01585428
2014	Nasopharyngeal Carcinoma Hepatocellular Carcinoma Breast Carcinoma	Phase I	Input of OKT3-activated TILs followed by interleukin 2 (IL-2) injection	ORR (80%)	NCT01462903
2015	Nasopharyngeal Carcinoma	Phase II	Cisplatin concurrent chemoradiotherapy (CCRT) combined with tumor-infiltrating lymphocyte (TIL)	ORR	NCT02421640
2017	Non-Small Cell Lung Cancer	Phase I	Cytoreductive chemotherapy with cyclophosphamide and fludarabine, TIL infusion, IL-2 treatment.	ORR(69%)	NCT03215810
2017	Cervical Carcinoma	Phase II	After the non-myeloablative (NMA) lymphocyte depletion regimen, autologous TIL were transfused (LN-145) followed by IL-2 therapy	ORR	NCT03108495
2017	Squamous Cell Carcinoma of the Head and Neck	Phase II	After NMA lymphocyte depletion, patients were infused with autologous TIL (LN-145) and then given IL-2.	ORR	NCT03083873
2019	Cervical Carcinoma	Phase I	Infusion of TILs after simultaneous radiotherapy with cisplatin	ORR(75%)	NCT04443296
2019	Metastatic Triple Negative Breast Cancer	Phase II	After NMA lymphocyte depletion, patients were infused with autologous TIL (LN-145) and then given IL-2.	ORR	NCT04111510
2019	Solid cancer	Phase II	Non-myeloablative lymphodepleting preparative regimen followed by infusion of autologous TIL and high-dose aldesleukin	ORR	NCT03935893
2019	Biliary Tract Cancer Cholangiocarcinoma Biliary Tract Neoplasms	Phase II	Intravenous push of TILs after a lymphocyte depletion preparation regimen consisting of fludarabine and cyclophosphamide	ORR	NCT03801083
2020	HCC	Phase I-II	Separate and infuse TILs.	Tolerability and safety	NCT04538313
2020	Metastatic or Recurrent Cervical Cancer	Phase I	Non-myeloablative (NMA) lymphocyte depletion regimen followed by infusion of autologous tumor-infiltrating lymphocytes (TIL) followed by infusion of IL-2	Tolerability and safety	NCT04674488
2021	Hepatobiliary-Pancreatic Cancers	Phase I	Input of TILs after NMA lymphodepletion treatment with hydroxychloroquine (600 mg, single dose) and cyclophosphamide	ORR	NCT05098197
2021	Epithelial Tumors	Early Phase I	Non-myeloablative lymphocyte depletion (NMA-LD) was treated with neoantigen to recognize TILs products, followed by IL-2	Tolerability and safety	NCT05141474
2021	Solid cancer	Early Phase I	Intravenous TILs after treatment with hydroxychloroquine (600 mg, single dose) and cyclophosphamide NMA lymphocyte depletion	Tolerability and safety	NCT05087745
2022	Refractory gynecologic cancers	Phase I-II	Infusion of autologous TIL followed by IL-2 administration after NMA lymphocyte depletion	tolerability and safety	NCT05152797
2022	Cervical Cancer	Phase I	Lymphocytes depleted after receiving C-TIL052A (autologous tumor-infiltrating lymphocytes, TIL) and IL-2 injections	Tolerability and safety	NCT05475847
2023	Solid cancer	Early Phase I	Input of TILs after treatment for non-clear myeloid lymphocyte depletion (LM103)	Tolerability and safety ORR	NCT05971576
2023	Solid cancer	Early Phase I	Input of TILs after NMA lymphocyte depletion followed by IL-2 administration	Safety and Efficacy	NCT05831033
2023	Solid cancer	Early Phase I	Input of TILs after treatment for non-clear myeloid lymphocyte depletion (LM103)	Tolerability and safety	NCT05971576

The primary bottleneck in hepatocellular TIL therapy lies in the identification and targeting of neoantigens, as well as the enrichment, expansion, and maintenance of antigen-specific T cells’ activity and clonogenicity. While traditional expansion methods may not suffice for achieving these goals, we can employ mutant gene screening to identify the TCRs of antigen-specific TILs and genetically engineer them for transcription into autologous T-lymphocytes. *TP53* is a frequently mutated gene in HCC and represents a potential immunotherapeutic target for this disease. Several studies have successfully transduced TCRs specific to *TP53* mutations into autologous lymphocytes, demonstrating promising efficacy and offering novel insights for the development of immunotherapy strategies against HCC (140).

## 5 Critical signaling pathways related to TILs in HCC

The occurrence and development of HCC is a complex process, often involving the aberrant activation of multiple signaling pathways. Previous studies have revealed a close association between the dysregulation of NF-κB, TGF-β, Wnt/β-Catenin, and JAK-STAT signaling pathways and the progression of HCC (16, 155, 156). The aberrant activation of the JAK-STAT signaling pathway contributes to hepatocyte development and progression (157). The NF-κB signaling pathway exerts a negative regulatory effect on the STAT3 signaling pathway, and ablation of STAT3 can effectively prevent the development of HCC (157). The aberrant activation of the Wnt/β-Catenin signaling pathway has been implicated in the metastasis of HCC (158, 159). The TGF-β signaling pathway exhibits a dual role in tumor biology, with early pathway activation primarily inhibiting tumor development while sustained aberrant activation of the late pathway promotes tumor progression (160).

### 5.1 TGF-β signaling pathway

**Figure 4:**
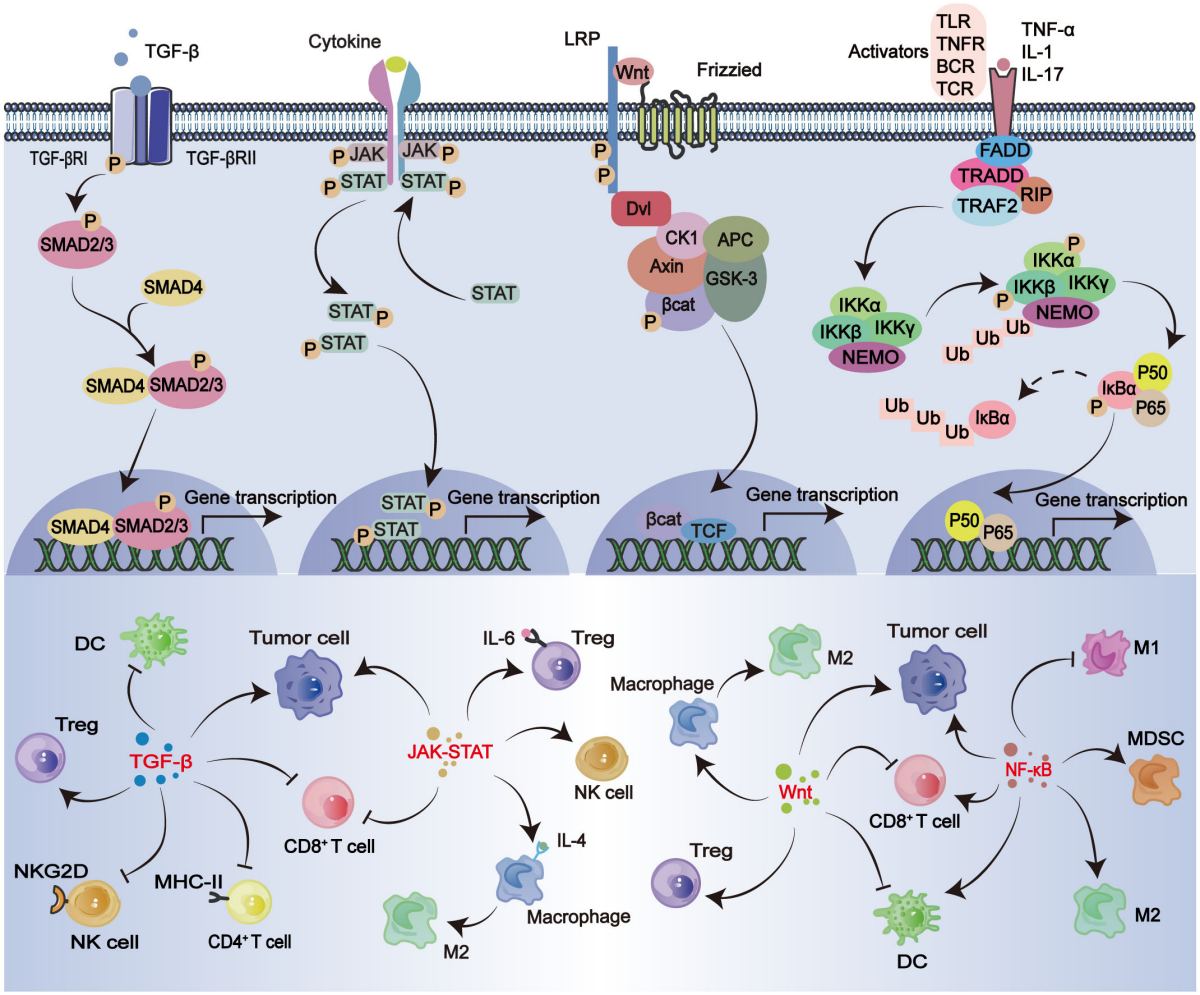
TGF-β signaling pathway. The TGF-β ligand family binds to type II and type I receptors on the cell membrane to form complexes. Phosphorylated type II receptors activate type I receptors to recruit and activate downstream SMAD2/3 proteins. SMAD4 interacts with SMAD2/3 proteins to form complexes, thereby inducing Smad proteins to aggregate in the nucleus. Transcriptional regulation as a transcription factor. TGF-β induces Tregs cell differentiation and promotes tumor progression. Inhibition of CD8^+^ T cells, CD4^+^ T cells, NK cells, DC infiltration. JAK-STAT signaling pathway. Cytokine binds to cell membrane surface receptor to activate JAK (Janus kinase) which activates the receptor, activated JAK binds and phosphorylates STAT protein, phosphorylated STAT protein forms a dimer, enters the nucleus and binds to DNA to regulate gene transcription. JAK-STAT signaling pathway induces Tregs differentiation, inhibits CD8^+^ T cell aggregation, affects sentence realization and cell transformation into M2 macrophages, and also promotes the survival of NK cells and DCs. Wnt/β-Catenin Signaling pathway. The Wnt protein binds to the Frizzled receptor on the cell membrane surface to form the Wnt-Frizzled complex. The activated Frizzled receptor interacts with the LRP to further activate the downstream signaling molecules. β-Catenin interacts with the complex and degrades and releases β-catenin, and the activated signaling molecule inhibits the activity of GSK-3β enzyme and prevents its phosphorylation of β-catenin, thereby stabilizing β-catenin. After the stable accumulation of β-Catenin enters the nucleus, it binds to the TCF/LEF transcription factor family and initiates the transcription of downstream target genes. Wnt/β-Catenin can inhibit the infiltration of CD8^+^ T cells and DCs, promote the differentiation of macrophages into M2 type and the infiltration of Tregs, and promote the development of tumors. NF - κB signaling pathway. Toll-like receptor (TLR), TCR, B cell receptor (BCR), tumor necrosis factor receptor (TNFR), and IL-1R stimulate NF-κB signaling. TNFR activation triggers TRADD aggregation, and TRAF2 interacts with protein kinase RIP1. RIP1 ubiquitinates and recruits NEMO. IκB is phosphorylated by the IKK complex, which labels iκbα by phosphorylation and subsequently degrades NF-κB (p50-p65) via a ubiquitin-dependent pathway and is transported to the nucleus. Inactivation of NF-κB signaling pathway can promote tumor progression and affect CD8^+^ T cells, and normal activation can promote the activation of CD8^+^ T cells and DCs. Overactivation increases the number of MDCS and also affects the transformation of macrophages from M2 phenotype to M1 phenotype.

TGF-β is a cytokine that governs diverse cellular processes, encompassing cell proliferation, differentiation, and immune responses (160). In the typical TGF-β signaling pathway, the TGF-β ligand family binds to type II and type I receptors on the cell membrane, forming complexes. Subsequently, phosphorylation of the type II receptor activates the type I receptor, which then recruits and activates downstream SMAD2/3 proteins (161, 162). This activation induces aggregation of Smad proteins in the nucleus for transcriptional regulation as transcription factors (161, 162). The interaction between SMAD4 and the SMAD2/3 protein leads to the formation of a complex that facilitates the transmission of TGF-β signals (**Figure 4**) (160). In the early stages of HCC, TGF-β exerts tumor-suppressive effects by inhibiting cellular proliferation and inducing cell cycle arrest. However, in advanced stages, dysregulation of the TGF-β signaling pathway occurs, resulting in the progression of HCC (163).

The dysregulated TGF-β signaling pathway in HCC can exert both direct and indirect effects on TILs. Within the tumor microenvironment, IFN-γ can induce the expression and secretion of TGF-β in HCC cells (**Figure 4**). Consequently, TGF-β directly inhibits the infiltration and activation of cytotoxic T cells, thereby impairing their anti-tumor activity (164, 165). The induction of Tregs differentiation also leads to the inhibition of effector T cell function and suppression of the anti-tumor immune response (166). The activities of SMAD, TGF-β receptor, and TGF-β can serve as indicators of the level of activation in the TGF-β signaling pathway. Nicotinamide nucleotide transhydrogenase-antisense RNA1(NNT-AS1) is found to be upregulated in HCC tissues, and its expression positively correlates with that of TGF-β, TGFβR1, and SMAD1/5/9 (164). This suggests that NNT-AS1 activates the TGF-β signaling pathway in HCC, leading to inhibition of CD4^+^ T cell infiltration and establishment of an immune escape mechanism (164). The expression of TGF-β induces the differentiation of tumor-infiltrating CD4^+^ naïve T cells into Tregs, thereby suppressing effector T cell function and inhibiting the anti-tumor immune response (166). The Tregs are capable of modulating the TGF-β signaling pathway, thereby promoting epithelial-mesenchymal transition (EMT) in HCC tumor cells, which is closely associated with hepatocyte metastasis (167). Cancer-associated fibroblasts (CAFs), which are present in the microenvironment of HCC, also belong to a subset of infiltrating immune cells. They have the ability to activate the TGF-β pathway, leading to the development of an immunologically compromised microenvironment in HCC (110). Chemokines secreted by CAFs, such as CCL2, CCL5, CCL7, and CXCL16, have been implicated in the progression of HCC (168). Specifically, CCL2 and CCL5 can activate the Hedgehog pathway, thereby promoting HCC migration. On the other hand, CCL7 and CXCL16 stimulate the activation of the TGF-β pathway in HCC, leading to metastasis (168). CXCL16 induces phosphorylation of SMAD2 and SMAD3 in HCC cells, thereby activating the TGF-β pathway. This direct activation of the TGF-β pathway has a significant impact on NK cell function (169). The DCs play a crucial role as aAPCs, and the suppression of MHC-II gene expression by TGF-β hampers the antigen-presenting capacity of DCs *in vitro*, thereby impacting the anti-tumor immune response (170). The secretion of TGF-β and IL-10 by MDSC in tumors has been demonstrated to inhibit the function of effector T cells and suppress antitumor responses (171, 172). The expression of Protein tyrosine kinase 7 (PTKP) is significantly upregulated in patients with advanced HCC, indicating its potential as a therapeutic target. PTKP enrichment enhances the metastatic potential of hepatocytes, which is regulated by the TGF-β pathway and SOXP activators (173).

### 5.2 JAK-STAT signaling pathway

The JAK-STAT pathway is a crucial signaling pathway involved in regulating normal cellular growth, survival, differentiation, and immune response (174, 175). In HCC, this pathway can become dysregulated due to genetic alterations or abnormal cytokine signaling, resulting in tumor progression (176). The binding of cytokines to cell membrane surface receptors triggers the activation of JAK (Janus kinase), which in turn activates the receptor, leading to its phosphorylation and subsequent binding and phosphorylation of STAT proteins (**Figure 4**) (177, 178). The resulting phosphorylated STAT proteins form dimers that translocate into the nucleus, where they bind to DNA and regulate gene transcription (177, 178).The JAK-STAT signaling pathway exerts direct or indirect regulation on various types of TILs within the microenvironment of HCC, thereby impacting their invasion, differentiation, apoptosis, cytotoxicity, and interaction. This pathway plays a crucial role in the development of HCC (**Figure 4**).

The expression of MEK inhibitors upregulates the JAK-STAT signaling pathway, thereby partially driving the upregulation of antigen-processing components (179). Consequently, this leads to an increased infiltration of CD8^+^ T cells in certain tumors and enhances their antitumor capacity (179). The secretion of IL-6 by HCC tumor cells, macrophages, and DCs is observed. The activation of the JAK-STAT signaling pathway mediated by IL-6 has been implicated in the pathogenesis of HCC (180). The activation of the stat signaling pathway by IL-6 may lead to the differentiation of CD73^+^γδ Tregs and subsequently impair the tumor-killing function of CD8^+^ T cells (181). The activation of the IL-6 signaling pathway in HCC enhances tumor infiltration of Tregs that suppress cytotoxic CD8^+^ T cells, thereby promoting immune evasion and tumor progression (182). The activation of the JAK-STAT signaling pathway in Native CD4^+^ T cells has been demonstrated to induce differentiation, proliferation, and suppression of anti-tumor responses in Th17 cells (183). Upon stimulation, dendritic cells secrete cytokines such as IL-12 to initiate the JAK-STAT signaling pathway (184). Subsequently, this activation of the JAK-STAT signaling pathway by dendritic cells facilitates the differentiation and maturation of Th1 cells, thereby promoting an effective immune response (184). Cytotoxic CD8^+^ T cells, NK cells, and other cell types have the capability to express chemokines CXCL9, CXCL10, and CXCL11 that are associated with T-cell helper type 1 function. Within the tumor microenvironment, stimulation of stat1 by IFN-γ influences the expression of Th1-type chemokines, leading to a reduction in the functional capacity of TILs (185). The secretion of IL-6 by fibroblasts in various tissues activates the JAK-STAT signaling pathway and upregulates the NOX4 gene, thereby promoting tumorigenesis and angiogenesis (blood vessel growth) (186). Other factors present in the tumor microenvironment, such as IL-4, activate the STAT3 signaling pathway in macrophages, leading to their transformation into M2 macrophages. These M2 macrophages can contribute to tumor development by directly or indirectly suppressing cytotoxic cell populations, including CD8^+^ T cells and NK cells (187–189). The activation of the JAK-STAT signaling pathway in M2 macrophages results in upregulated expression of cytokines PIM1 and VEGF-α, thereby promoting hepatocyte proliferation and metastasis (190). The activation of NK cells in HCC is associated with the STAT signaling pathway in HCC cells. Blocking IL-6-stimulated STAT3 activation in these cells may lead to alterations in cytokines within the tumor microenvironment. The consequence of this is a reduction in the secretion of TGF-β and IL-10, an increase in type I interferon levels (which activates NK cells, DCs, CD4^+^ T cells, and CD8^+^ T cells), stimulation of NK cells, and augmentation of anti-tumor immunity (191, 192). When the JAK-STAT pathway is dysregulated in HCC cells, immunosuppressive cytokines such as IL-10 and TGF-β may be produced to inhibit the function of TILs and impair their ability to infiltrate and attack HCC cells, thereby facilitating tumor immune evasion (171, 193, 194).

### 5.3 Wnt/β-catenin signaling pathway

The Wnt/β-catenin signaling pathway is a complex and evolutionarily conserved signaling cascade that plays crucial roles in cellular development, proliferation, apoptosis, and other physiological processes, thereby exerting profound effects on fundamental biological functions (195, 196). The Wnt protein binds to the Frizzled receptor on the cell membrane surface, forming the Wnt-Frizzled complex and inducing conformational changes (197). Subsequently, the activated Frizzled receptor interacts with LRP to further initiate downstream signaling involving molecule (197). The degradation of β-Catenin is associated with a complex consisting of kinases CK1 and GSK3, as well as suppressor proteins AXIN and APC (198). The dishevelled protein (DVL) mediates the interaction between the Frizzled receptor and a complex, facilitating the release of β-catenin from degradation (198–200). The activated signaling molecule inhibits the activity of the GSK-3β enzyme, thereby preventing its phosphorylation of β-catenin and facilitating the stabilization of β-catenin (197). The stable accumulation of β-Catenin within the nucleus leads to its binding with the TCF/LEF transcription factor family, thereby initiating downstream target gene transcription (**Figure 4**) (197). The dysregulation of the Wnt/β-Catenin signaling pathway in hepatocellular cancer cells may contribute to the progression of early-stage HCC (201–204). The arid nature of HCC is a prominent contributor to the recurrence and drug resistance of liver cancer, with metastasis occurring predominantly in the advanced stages of the disease, which can be attributed to aberrant activation of the Wnt/β-Catenin signaling pathway (205–209). KIAA1522 exhibits high expression levels in HCC and activates the Wnt/β-Catenin signaling pathway, thereby promoting the progression of HCC (201). Aberrant activation of the Wnt/β-catenin cascade has been observed at the genetic level in 30% to 40% of HCC cases, with elevated Wnt expression being associated with a poorer prognosis for HCC (210, 211).

The activation of the Wnt/β-Catenin signaling pathway in HCC is closely associated with the infiltration of TILs (**Figure 4**). Excessive activation of this pathway can impact both T cell infiltration and their anti-tumor effects, ultimately leading to immune evasion by the tumor. Studies have demonstrated that the Wnt/β-Catenin signaling pathway exerts inhibitory effects on the anti-tumor activity of TILs through two distinct mechanisms: 1. modulating the functionality and differentiation of Tregs. 2. influencing the infiltration of CD8^+^ T cells into tumors (212, 213). The sBBI&PDP nanoparticles, consisting of racemic spherical supramolecular peptides, effectively suppressed the hyperactive Wnt/β-Catenin signaling pathway while simultaneously inhibiting endogenous PD-L1 expression (214). This dual action resulted in enhanced infiltration and activation of CD8^+^ T cells within the tumor microenvironment (214). Not only that, the Wnt/β-Catenin signaling pathway in the tumor microenvironment exerts a significant impact on various types of TILs. The Wnt/β-Catenin signaling pathway plays a crucial role in the development and function of DCs, with CD103^+^ DCs serving as a pivotal subset within the DC population that drives effector T cell responses and contributes to the recruitment of effector T cells (215, 216). However, activation of the Wnt/β-Catenin signaling pathway leads to a reduction in CD103^+^ DCs, which are crucial for recruiting effector T cells through the release of chemokines CXCL9 and CXCL10. Consequently, the Wnt/β-Catenin signaling pathway hampers effector T cell infiltration into tumor tissues, thereby favoring continued tumor development (215, 216). The activation of β-Catenin in HCC cells directly impacts the infiltration of DCs (217). The differentiation of macrophages in the tumor microenvironment is also regulated by the Wnt/β-Catenin signaling pathway. Macrophage-derived Wnt ligands activate Wnt signaling in tumor cells, and the Wnt signaling produced by tumor cells also influences macrophage differentiation (218, 219). The evidence suggests that Wnt ligands secreted by tumor cells facilitate the activation of the macrophage Wnt/β-Catenin signaling pathway, thereby inducing polarization of macrophages towards an M2-like phenotype and ultimately promoting tumor growth (218, 219). The expression of LINC00662 in HCC exhibited an upregulation trend, activating Wnt/β-catenin signaling by inducing the secretion of Wnt3. This activation inhibited apoptosis and promoted invasion of HCC cells (219). The association between high expression of Wnt3 and low infiltration of CD8^+^ T cells in HCC has been demonstrated (211). The infiltration of Tregs cells in tumor tissue promotes the progression of HCC, and their secretion of negative immunomodulatory factors (Foxp3, TGF-β, and IL-10) is associated with activation of the Wnt/β-catenin signaling pathway (220). The coexistence of TCF-1 and Foxp3 imposes a constraint on the expression of Tregs, while activation of the Wnt/β-catenin signaling pathway disrupts this constraint and facilitates disease progression (221).

### 5.4 NF-κB signaling pathway

The NF-κB signaling pathway is crucial in regulating immune response and inflammation, and its dysregulation has been linked to various diseases, including cancer (222). The NF-κB pathway is a highly conserved signaling pathway, and its aberrant activation plays a crucial role in tumorigenesis and progression. Among the canonical pathways that activate the NF-κB signaling pathway, stimulation of Toll-like receptor (TLR), T-cell receptor (TCR), B-cell receptor (BCR), tumor necrosis factor receptor (TNFR), and IL-1 receptor (IL-1R) have been shown to activate NF-κB signaling (223, 224). The activation of TNFR triggers the aggregation of TRADD, the interaction between TRAF2 and protein kinase RIP1, the ubiquitination of RIP1, and the recruitment of NEMO (225–227). The IKK complex (composed of two kinases, IKKa and IKKb, and a regulatory subunit, NEMO) phosphorylates IκB to label IκBα for degradation via a ubiquitin-dependent pathway. NF-κB (p50-p65) is then translocated into the nucleus (**Figure 4**) (228, 229). The NF-κB signaling pathway in HCC cells also influences intracellular cholesterol levels, leading to pro-inflammatory effects on cholesterol (230). The activation of the NF-κB signaling pathway in HCC cells is associated with HCC angiogenesis, tumor growth, and metastasis. IKKβ-dependent classical NF-κB signaling regulates cell survival, immunity, and inflammation, influencing HCC progression (231–233).

The NF-κB signaling pathway not only impacts HCC cells themselves but also plays a crucial role in the recruitment, inactivation, antitumor effects, and interactions of TILs (**Figure 4**). The initial activation of primitive T cells through TCR and co-stimulatory signaling relies on the canonical NF-κB pathway (234). Inhibiting the NF-κB pathway in T cells hampers their differentiation into Th1 cells (235). The anti-tumor effects of CD8^+^ T cells are linked to the activation of the NF-κB signaling pathway, and studies have demonstrated that impaired activation of this pathway leads to reduced responsiveness in CD8^+^ T cells (236). The down-regulation of TPX2 expression in tumor-infiltrating CD8^+^ T lymphocytes in HCC leads to the inactivation of the NF-κB signaling pathway, resulting in a weakened anti-tumor effect of these lymphocytes (237). The activation of the NF-κB signaling pathway in TAM and MDSCs induces the expression of pro-inflammatory factors (IL-10) and chemokines (CCL17, CCL2), while inhibiting the expression of inflammatory cytokines (TNF α, IL-1, IL-6, etc.), leading to M2 macrophage activation and tumor evasion (238, 239). The inhibition of the NF-κB signaling pathway in TAMs shifts their phenotype from a tumor-promoting M2 state to an m1-like cytotoxic state, thereby facilitating tumor regression (240). In addition, activation of the NF-κb signaling pathway in hepatocellular cancer cells leads to CCL9 secretion and promotes TAM recruitment (241). The hyperactivation of the WDR6/UVRAG/NF-κB pathway in HCC cells leads to an increase in the population of MDSCs within the tumor and a decrease in the infiltration of CD8^+^ T lymphocytes, thereby promoting HCC progression (242). The maturation of DCs was associated with the activation of the NF-κB signaling pathway induced by 4-1BB co-stimulation, which also promoted the growth of tumor-specific CD8^+^ TILs (243). In hepatocytes, activation of the NF-κB signaling pathway inhibits apoptosis and contributes to tumor development (244).

## 6 Discussion

The presence of TILs in HCC can exert a significant impact on its progression. For instance, CD8^+^ T cells, NK cells, and B cells have been shown to possess inhibitory effects on tumor progression, whereas Tregs, MDSCs, and macrophages contribute to the formation of an immunosuppressive microenvironment. The efficacy of TIL may be influenced by multiple factors, including the tumor’s mutational burden and microenvironment, the diversity of TCR libraries, the functionality and abundance of TILs themselves, the differentiation state of TILs, the presence of antitumor phenotypes, and the clonability within the tumor.

TIL, which possess tumoricidal activity, specifically recognize neoantigens unique to tumors. The infiltrative capacity of TIL is influenced by the number of tumor-induced mutations, thereby impacting therapeutic efficacy (245). For instance, malignancies characterized by a higher mutational burden, such as melanoma, exhibit an augmented repertoire of neoantigens for immune recognition and consequently demonstrate superior prognoses compared to breast cancers with a lower mutational load (145). Antigen-specific TILs may exhibit a state of depletion and encounter antigen loss during the amplification process, or demonstrate limited clonogenic capacity following infusion, thereby impeding the generation of a sustained immune response (246). The stimulation factors related to tumor microenvironment also affect the activation state of TIL (247). To tackle the challenge of targeting neoantigens with TIL and enhancing their ability to induce a sustained immune response *in vivo*, researchers conducted a screening process to identify TCRs reactive against neoantigens (137). Subsequently, these TCRs were genetically engineered for transcription into autologous lymphocytes, aiming to enrich for reactive TIL and enhance therapeutic efficacy (137). Efforts are underway to develop technologies for targeting neoantigen-specific T cells through the sorting of multiple surface markers (148). Considering the immunological characteristics of HCC, we also propose the potential application of genetically modifying *TP53* mutation-responsive TCR into PBL for therapeutic purposes.

Dysregulated TGF-β signaling in HCC can result in immune evasion and tumor progression. Within the tumor microenvironment, it exerts direct or indirect effects on the infiltration of TILs. The TGF-β signaling pathway contributes to impaired activation of cytotoxic T cells, enhanced activation of Tregs, and reduced activation of NK cells and DCs, thereby influencing the progression of HCC. Enhancing our understanding and modulation of TGF-β signaling pathways while harnessing the potential of TILs is anticipated to enhance therapeutic outcomes for HCC.

The JAK-STAT signaling pathway plays a pivotal role in the regulation of HCC progression, impacting the infiltration of TILs, including T-lymphocytes and NK cells, within tumor tissues. Moreover, it exerts influence on the differentiation and synergistic anti-tumor effects of other immune cells such as macrophages and dendritic cells. However, further investigation is warranted to comprehensively elucidate the intricate interactions between the JAK-STAT pathway and TILs in HCC, as well as to develop efficacious therapeutic strategies that harness these interactions for improved patient prognosis.

Aberrant activation of the Wnt/β-Catenin signaling pathway in HCC profoundly impacts the growth and differentiation of diverse subsets of TILs, exerting direct or indirect effects on metastasis, immune evasion, and HCC development (248). Dysregulated Wnt/β-Catenin signaling also disrupts dendritic cell activation and antigen presentation, diminishes effector T-cell infiltration, and promotes macrophage polarization towards the M2 phenotype to facilitate tumor progression.

The interplay between NF-κB signaling pathway and TILs is implicated in inflammation-associated HCC progression. Moreover, NF-κB signaling modulates M1/M2 macrophage differentiation as well as T lymphocyte differentiation and infiltration. Given the intricate crosstalk among NF-κB signaling, TILs, and the tumor microenvironment in HCC pathogenesis, ongoing investigations are exploring potential therapeutic strategies targeting these pathways. These approaches encompass immunotherapies aimed at augmenting TIL function, inhibitors of NF-κB signaling to attenuate inflammatory and pro-tumorigenic signals, as well as combination therapies concurrently modulating both pathways.

Previous research has confirmed that the addition of TGF-β during the laboratory culture phase can increase the percentage of CD8^+^ T cells in TIL products, enhancing their anti-tumor activity (128). Our study also summarizes how the activation of specific signaling pathways can affect the state of infiltrating TILs in HCC, thereby influencing the disease outcome. Building on this, we anticipate that incorporating factors related to these signaling pathways during the TIL cultivation phase for HCC might regulate these pathways and impact the TIL product, offering new perspectives for the cultivation of HCC in TILs. Exploring the mechanisms underlying the impact of dysregulated signaling pathways on different types of TILs and their direct or indirect effects on HCC opens up new avenues for research. The combination of signaling pathways and TILs for the management of HCC may emerge as a promising therapeutic approach in future studies.

## Author contributions

XiaW: Conceptualization, Writing – review & editing, Investigation, Visualization, Writing – original draft. ZY: Investigation, Visualization, Writing – original draft, Writing – review & editing. ZL: Investigation, Visualization, Writing – original draft. XH: Investigation, Writing – review & editing. YiZ: Visualization, Writing – review & editing. XinW: Writing – review & editing. JSu: Writing – review & editing. XuW: Writing – review & editing. ML: Writing – review & editing. FD: Writing – review & editing. YC: Writing – review & editing. SD: Writing – review & editing. YuZ: Investigation, Writing – review & editing. JSh: Writing – review & editing. TY: Conceptualization, Supervision, Writing – review & editing. ZX: Conceptualization, Funding acquisition, Supervision, Writing – review & editing.
